# Chemical Composition, In Vitro Digestibility and Rumen Fermentation Kinetics of Agro-Industrial By-Products

**DOI:** 10.3390/ani9110861

**Published:** 2019-10-24

**Authors:** Jairo García-Rodríguez, María José Ranilla, James France, Héctor Alaiz-Moretón, María Dolores Carro, Secundino López

**Affiliations:** 1 Departamento de Producción Animal, Universidad de León, E-24007 León, Spain; jgarr@unileon.es; 2 Instituto de Ganadería de Montaña, CSIC-Universidad de León, Finca Marzanas s/n, 24346 Grulleros, Spain; 3 Department of Animal Biosciences, University of Guelph, Guelph, ON N1G 2W1, Canada; jfrance@uoguelph.ca; 4 Departamento de Ingeniería Eléctrica de Sistemas y Automática, Escuela de Ingeniería Industrial e Informática, Universidad de León, Campus Universitario de Vegazana, 24071 León, Spain; hector.moreton@unileon.es; 5 Departamento de Producción Agraria, Escuela Técnica Superior de Ingeniería Agronómica, Agroalimentaria y de Biosistemas, Universidad Politécnica de Madrid, Ciudad Universitaria, 28040 Madrid, Spain; mariadolores.carro@upm.es

**Keywords:** ruminant, feedstuff, nutritive value, digestibility, gas production technique, multivariate data analysis, hierarchical clustering

## Abstract

**Simple Summary:**

Agro-industrial by-products are the waste from either agricultural crops or vegetable processing industries, and their disposal represents an environmental problem since they are potential pollutants. One of their most promising alternative uses is as feedstuffs in ruminant diets. The aim of this study was to assess the nutritive value of different by-products by analysing their chemical composition, in vitro digestibility and gas production kinetics. The results showed a high variability in chemical composition, in vitro digestibility and rumen fermentation kinetics among different by-products. In addition, samples of the same by-product from a different origin or subjected to different conservation processes showed a certain variability in the evaluated parameters. The variability among by-products was reflected in the results of the cluster analysis, which divided the materials in four groups based on the multivariate analysis. Most by-products showed the potential to be included as alternative ingredients in ruminant rations. They could be used as a source of energy, fibre or protein, replacing part of the ingredients in a conventional diet and therefore, reducing the risk of environmental pollution and contributing to develop a circular economy by recycling these wastes. In addition, their use as feedstuffs might reduce competition between ruminants and humans for food or land.

**Abstract:**

The nutritive value of 26 agro-industrial by-products was assessed from their chemical composition, in vitro digestibility and rumen fermentation kinetics. By-products from sugar beet, grape, olive tree, almond, broccoli, lettuce, asparagus, green bean, artichoke, peas, broad beans, tomato, pepper, apple pomace and citrus were evaluated. Chemical composition, in vitro digestibility and fermentation kinetics varied largely across the by-products. Data were subjected to multivariate and principal component analyses (PCA). According to a multivariate cluster analysis chart, samples formed four distinctive groups (A–D). Less degradable by-products were olive tree leaves, pepper skins and grape seeds (group A); whereas the more degradable ones were sugar beet, orange, lemon and clementine pulps (group D). In the PCA plot, component 1 segregated samples of groups A and B from those of groups C and D. Considering the large variability among by-products, most of them can be regarded as potential ingredients in ruminant rations. Depending on the characteristic nutritive value of each by-product, these feedstuffs can provide alternative sources of energy (e.g., citrus pulps), protein (e.g., asparagus rinds), soluble fibre (e.g., sugar beet pulp) or less digestible roughage (e.g., grape seeds or pepper skin).

## 1. Introduction

The world’s human population is in constant growth, and estimated to reach 8.6 billion people by 2030 and 9.8 billion by 2050 [1]. Consequently, there is a necessity to increase food production, from both plant and animal origins. Fresh vegetables and fruit production in Spain reached nearly 38,600 hundred tons in 2017 (including olives and grapes) [2], so that Spain is the largest vegetable and fruit producing country in the European Union (EU) [3]. Throughout the plant-based food supply chain there is a loss or waste of material generated during the harvesting or processing of food for human consumption. These wastes are known as by-products and their disposal may represent an environmental problem, as they are perishable and potential pollutants [4]. These by-products can have different applications or uses. One valuable possibility in the field of animal nutrition is the recycling of these materials as alternative feedstuffs, in particular for herbivores [5,6]. Ruminants can digest plant fibre to produce high quality products available to humans such as milk or meat [7]. The potential use of these by-products as ingredients in ruminant diets could reduce the environmental impact of the disposal of these residues [6], and would favour of the development of a circular economy by recycling the biomass derived from crop production. Also, the use of these by-products as ruminant feedstuffs could reduce competition between ruminants and humans (or other non-ruminant farm animals) for food or land, contributing to moderate the demand for feed resources (cereals or forage) by these species [8].

The use of agro-industrial by-products as animal feeds has been a matter of interest for the last few decades [6,9]. However, there is a high variability in their chemical composition associated with multiple factors, such as their botanical or geographical origin, treatments during harvesting and processing, or the climatic conditions during their cultivation, among others [10]. This variability offers a wide range of potential ingredients that could partially or totally substitute for different feeds in ruminant rations. By-products with high energy content could replace the grains in the ration [11,12,13], whereas those with high fibre content might replace the roughage [14,15]. Also, some by-products rich in nitrogen could be an alternative to protein supplements [16,17]. Therefore, there is a need for information on the chemical composition and nutritive value of materials and by-products derived from agriculture, horticulture and fruit farming to expand our knowledge on their potential as feedstuffs before making recommendations about their inclusion in ruminant diets.

Chemical composition, in vitro digestibility and in vitro rumen fermentation kinetics are useful tools for feed evaluation. Compared to in vivo experiments, in vitro methods are cheaper and allow for a more precise control of experimental conditions and for the screening of a large number of materials in a relatively short time [18,19]. The large variability among by-products in chemical composition and nutritive value for ruminants represents an important issue, making it difficult to establish general recommendations on their use as animal feed. This study was designed to determine the chemical composition, in vitro digestibility and fermentation kinetics of a wide range of agro-industrial by-products as an indicator of their potential use as feedstuffs for ruminants.

## 2. Materials and Methods

In total, 26 agro-industrial by-products were used in this study, namely sugar beet pulp (either dehydrated or ensiled), sugar beet tops (including leaves), beet root leftovers (including rootlets, hairs, root tips and beet tails), grape seeds, olive tree leaves, almond hulls, broccoli stalk (hay), lettuce leaves, asparagus rinds (two samples from different origin), green bean haulms (either alone or mixed with sugar beet pulp), artichoke by-product (either fresh or ensiled), pea haulms, broad bean haulms, dried tomato pulp, pepper cores, pepper skin, apple pomace (either alone or mixed with pear pomace) and citrus pulps (one from lemon, one from clementine and two orange pulps from different origin). Representative samples of each by-product were collected from vegetable canning and other agro-food industries located in different places across Spain (León, La Rioja, Navarra, Zaragoza and Murcia). Table 1 shows the geographical origin of samples.

Chemical composition of the by-products was analysed in the laboratory on oven-dried samples (55 °C) to determine dry matter (DM), and then ground through a 1-mm screen. Determination of organic matter (OM), ether extract (EE) and crude protein (CP) followed the methods of the Association of Official Analytical Chemists (AOAC) [20]. Neutral detergent fibre (NDF), acid detergent fibre (ADF) and acid detergent lignin (ADL) contents were determined according to Van Soest et al. [21], using an ANKOM220 Fiber Analyzer unit (ANKOM Technology Corporation, Fairport, NY, USA).

In vitro digestibility was carried out with all by-products as described by Goering and Van Soest [22] with some modifications [23], using an DAISY^II^ incubator, digestion jars and the filter bag technology proposed by ANKOM (Macedon, NY, USA). Four rumen cannulated Merino sheep were used to obtain rumen fluid for the in vitro incubations. Animals were housed in individual pens and fed alfalfa hay (1 kg/day) with free access to water and mineral/vitamin licks. Sheep were cared and handled by trained personnel in accordance with the Spanish guidelines for experimental animal protection (Spanish Royal Decree 53/2013 on the protection of animals used for experimentation or other scientific purposes). The experimental protocols were approved by the Institutional Ethics Committee on Animal Experimentation (ULE_014_2016) of Universidad de León and the Junta de Castilla y León (Spain). Rumen contents were withdrawn before morning feeding and were strained through four layers of cheesecloth, collected into pre-warmed thermal flasks with an O_2_ free headspace, and kept at 39 °C under a CO_2_ atmosphere. Samples of by-products (250 mg) were weighed into artificial fibre bags (5 × 5 cm ANKOM F57 filtering bags, pore size 20 µm), which were heat sealed and introduced into 5 L incubation jars (24 bags per jar). Filtered rumen fluid was diluted in the culture medium of Goering and Van Soest [22] in the proportion 1:4 (v/v). Then, diluted (buffered) rumen fluid (2 L) was anaerobically transferred into the incubation jars and closed with a plastic lid with a single-way valve to prevent the accumulation of fermentation gases. Jars were placed in a revolving incubator (ANKOM Daisy Incubator, ANKOM Technology Corp, Macedon, NY, USA) where they were maintained at 39 °C with continuous rotation to ensure the correct immersion of the bags in buffered rumen fluid. Incubation lasted 48 h and then bags were rinsed gently in cold water, oven-dried at 60 °C and weighed to calculate in vitro DM digestibility. After that, bags were introduced in the ANKOM Fiber Analyzer and subjected to a neutral detergent extraction at 100 °C for 1 h according to Van Soest et al. [21] to determine in vitro NDF digestibility. Two incubation runs were carried out with duplicate determinations for each feedstuff in each run, thus giving four observations per by-product.

The in vitro gas production (GP) technique proposed by Theodorou et al. [24] was used to assess the rumen fermentation kinetics of the by-products. Rumen fluid was diluted (1:4 v/v) with the culture medium of Goering and Van Soest [22]. Medium preparation and the addition of rumen fluid were carried out under continuous flushing with CO_2_. Ground samples (500 mg) were mixed with 50 mL of diluted rumen fluid and incubated at 39 °C in 120 mL culture bottles under a CO_2_ atmosphere. Bottles were sealed with rubber stoppers and aluminium caps. In each run, four bottles were incubated as blanks. The GP was measured at several incubation times (3, 6, 9, 12, 16, 20, 24, 30, 36, 48, 60, 72, 96, 120 and 144 h after inoculation time), using a pressure transducer and a calibrated syringe [24]. After 144 h, contents of each bottle were filtered using glass filter crucibles under vacuum. The incubation residue after 144 h was oven-dried at 55 °C for 48 h to determine potential DM disappearance (D144). Three incubation runs were conducted in three consecutive weeks giving a total of three determinations for each substrate. Metabolizable energy (ME, MJ/kg DM) content was estimated using CP and EE contents (in g/kg DM) and the volume of gas measured after 24 h of incubation (G24 in mL per 200 mg DM incubated) as described by Menke and Steingass [25]:

(1) ME= 2.43+0.1206× G24+0.0069×CP+0.0187×EE.

To estimate fermentation kinetics parameters, data on cumulative gas production were fitted using the exponential model proposed by France et al. [26]:

(2) $$G=A\;\;\left[1-e^{-c\;\left(t-L\right)}\right]$$

where *G* (mL) represents cumulative gas production at time *t*, *A* (mL) is the asymptotic gas production, *c* (h^−1^) is the fractional fermentation rate and *L* (h) is lag time. The half-life (*t*_1/2_, h) of the degradable fraction of each substrate was calculated as the time taken for gas accumulation to reach 50% of its asymptotic value. The partitioning factor (PF) was calculated as mg DM digested (D144)/mL fermentation gas (*A*) [27] as an indicator of the efficiency of ruminal fermentation.

The extent of degradation of each by-product in the rumen (*E*, g DM degraded/g DM ingested) for a given rate of passage (*k*, h^−1^) was estimated following the approach derived by France et al. [26]. To calculate *E*, a mean retention time of digesta in the rumen of 30 h was assumed, giving a rate of passage of 0.033 h^−1^ (which can be found in sheep fed on a forage diet at maintenance level).

Multivariate hierarchical cluster analysis [28] was performed using the chemical composition, in vitro digestibility and gas production kinetics data to group the by-products. The method used for hierarchical agglomerative classification was complete linkage clustering based on a furthest neighbour criterion, with the furthest pair of observations between two groups used to determine (dis)similarity of the two groups [28]. The similarity and dissimilarity measures were calculated as squared Euclidean distances. The chart shows the distances between clusters. The similarity between them was calculated as 100—distance. The SAS System for Windows was used for cluster analysis (SAS software, Version 9.1; Copyright © 2002–2003 SAS Institute Inc. Cary, NC, USA).

## 3. Results

### 3.1. Chemical Composition Analysis

Chemical composition and ME content of by-products are shown in Table 2. Predictably, chemical composition was highly variable between by-products. The OM content varied from 769 (green beans haulms) to 987 g/kg DM (apple and pear pomace), although only three samples had an OM content below 850 g/kg DM (olive tree leaves, lettuce leaves and green beans haulms). CP varied widely, ranging from 48 (apple and pear pomace) to 226 g/kg DM (asparagus rinds 2). EE content was also variable, with values from 3 (sugar beet pulp) to 77 g/kg DM (lemon pulp). In the same way, NDF content varied widely, from 139 (clementine pulp) to 753 g/kg DM (pepper skins), ADF from 96 (clementine pulp) to 641 g/kg DM (pepper skins), and ADL from 2 (lemon and orange pulp 1) to 437 g/kg DM (grape seeds). A large variation among by-products was observed in the estimated ME content, ranging from 4.4 (pepper skins) to 12.6 MJ/kg DM (lemon pulp).

### 3.2. In Vitro Gas Production and Fermentation Kinetics

Results from in vitro gas production kinetics are shown in Table 3. As with chemical composition, values from the evaluated parameters varied between substrates. The asymptotic gas production values (*A*) ranged from 78 (grape seeds) to 374 mL gas/g DM (orange pulp 1). It should be noted that *A* was less than 200 mL gas/g DM for only three substrates (grape seeds, pepper skins and olive tree leaves). Lemon pulp had the highest fractional rate of fermentation (*c*) value (0.090 h^−1^), while pepper skins had the lowest (0.036 h^−1^). Regarding lag time, values varied from 0 h (grape seeds) to 4.60 h (artichoke by-product hay). Gas produced at 24 h (G24) ranged between 62 (grape seeds) and 314 mL gas/g DM incubated (orange pulp 1). Average gas production rate (AR) varied from 2.9 (pepper skins) to 19.2 (lemon pulp) mL gas/g DM per h. The lowest *t*_1/2_ value corresponded to lemon pulp (9 h) and the highest to pepper skins (22 h). Finally, PF values ranged between 2.39 (apple and pear pomace) and 4.06 (lettuce leaves) mg DM digested/mL.

### 3.3. In Vitro Digestibility

The in vitro digestibility results are shown in Table 4. In vitro DM digestibility (IVDMD) coefficients ranged from 0.362 (grape seeds) to 0.991 (clementine pulp), although only three by-products had values below 0.600 (grape seeds, pepper skins and apple pomace). In vitro NDF digestibility (IVNDFD) followed a similar pattern, with a wide range for the different by-products. IVNDFD coefficients varied from 0.064 (grape seeds) to 0.949 (orange pulp 1). Notwithstanding, more than half of the by-products yielded IVNDFD values above 0.600. Predictably, DM disappearance after 144 h of incubation (D144) varied from 0.227 (grape seeds) to 0.979 g/g DM incubated (orange pulp 1). Finally, the extent of degradation (*E*) was also variable between substrates, ranging from 0.150 (grape seeds) to 0.667 g DM degraded/g DM ingested (lemon pulp).

### 3.4. Cluster Analysis

**Figure 1:**
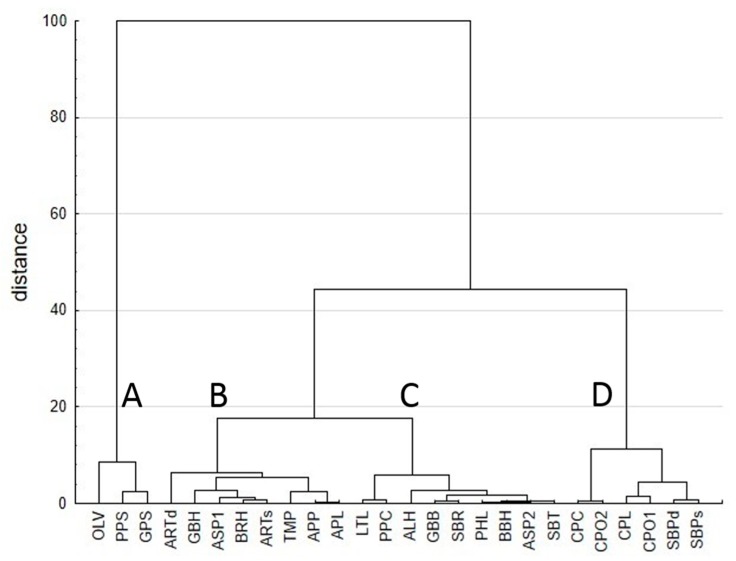
Multivariate cluster analysis plot showing groups of different by-products based on similarities in chemical composition, in vitro digestibility and fermentation kinetics: OLV (olive tree leaves), PPS (pepper skins), GPS (grape seeds), ARTd (hay of artichoke by-products), GBH (green bean haulms), ASP1 (asparagus rinds 1), BRH (broccoli stalk hay), ARTs (ensiled artichoke by-products), TMP (dried tomato pulp), APP (apple and pear pomace), APL (apple pomace), LTC (lettuce leaves), PPC (pepper cores), ALH (almond hulls), GBB (green bean haulms and sugar beet pulp), SBR (sugar beet rootlets, hairs, root tips and beet tails), PHL (pea haulms), BBH (broad bean haulms), ASP2 (asparagus rinds 2), SBT (sugar beet tops and leaves), CPC (clementine pulp), CPO2 (orange pulp 2), CPL (lemon pulp), CPO1 (orange pulp 1), SBPd (sugar beet pulp dehydrated) and SBPs (ensiled sugar beet pulp).

Results of cluster analysis are shown in Figure 1. The chart shows that samples are classified in four clusters (A–D) with a level of similarity among by-products within each group greater than 90%. The most distinctive group (A) included the least degradable/digestible by-products (olive tree leaves, pepper skins and grape seeds). On the contrary, the cluster D grouped the most degradable by-products (clementine, orange and lemon pulps, dehydrated and ensiled sugar beet pulps). In the middle (between groups A and D), groups B and C represented those by-products with an intermediate degradability/digestibility. Group C included lettuce leaves, pepper cores, almond hulls, mixture of green bean haulms and sugar beet pulp, sugar beet rootlets, pea haulms, broad bean haulms, asparagus rinds 2 and sugar beet tops and leaves. Group B included the rest of the by-products, namely artichoke by-products hay, green bean haulms, asparagus rinds 1, broccoli stalk hay, artichoke by-products silage, tomato pomace, apple and pear pomace and apple pomace. These latter groups (B and C) merged at a level of similarity of 82%, and the joint cluster B + C showed a similarity of 55% with group D, merging in a macro cluster (B + C + D) clearly discriminated from group A.

### 3.5. Principal Components Analysis (PCA)

**Figure 2:**
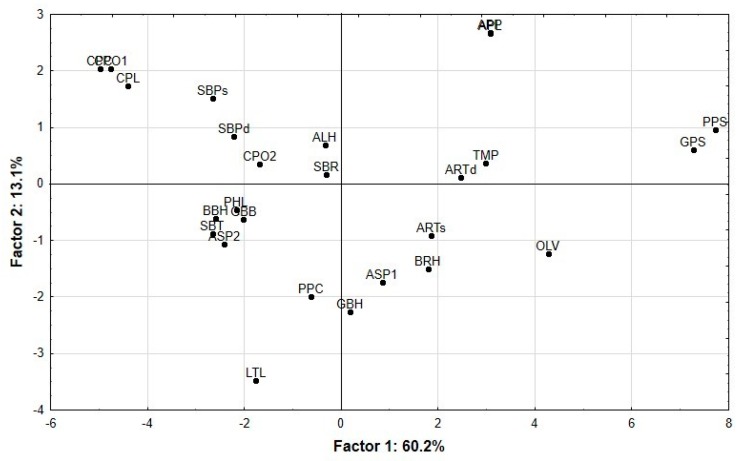
Principal component analysis showing the grouping and separation of the by-products based on chemical composition, in vitro digestibility and fermentation kinetics: OLV (olive tree leaves), PPS (pepper skins), GPS (grape seeds), ARTd (hay of artichoke by-products), GBH (green bean haulms), ASP1 (asparagus rinds 1), BRH (broccoli stalk hay), ARTs (ensiled artichoke by-products), TMP (dried tomato pulp), APP (apple and pear pomace), APL (apple pomace), LTC (lettuce leaves), PPC (pepper cores), ALH (almond hulls), GBB (green bean haulms and sugar beet pulp), SBR (sugar beet rootlets, hairs, root tips and beet tails), PHL (pea haulms), BBH (broad bean haulms), ASP2 (asparagus rinds 2), SBT (sugar beet tops and leaves), CPC (clementine pulp), CPO2 (orange pulp 2), CPL (lemon pulp), CPO1 (orange pulp 1), SBPd (sugar beet pulp dehydrated) and SBPs (ensiled sugar beet pulp).

Results of PCA are shown in Figure 2. Components 1 and 2 explained 60.2% and 13.1% of the variance, respectively. Loadings of each variable used in the PCA on principal components 1 and 2 explained the distribution of the samples in the plot. Samples with high NDF, ADF and ADL contents and *t*_1/2_ values were positioned on the right of the plot, but the ones with high values of in vitro digestibility (IVDMD and IVNDFD), fermentation kinetics parameters (*A*, *c*, G24, AR, D144 and *E*), and non-structural carbohydrates (NSC) and ME contents were positioned on the left along the x-axis. By-products with high CP content and PF were at the bottom of the plot, whereas those with high OM content and Lag were at the top. Principal component 1 clearly separated the samples. Citrus pulps (clementine, lemon and orange 1 and 2), sugar beet by-products (dehydrated or ensiled sugar beet pulp, sugar beet rootlets and sugar beet tops and leaves), almond hulls, pepper cores, lettuce leaves, pea haulms, broad bean haulms, asparagus rinds 2 and the mixture of green bean haulms and sugar beet pulp were on the left the axis. In contrast, the other by-products (including olive tree leaves, pepper skins, grape seeds, dried tomato pulp, broccoli stalk hay, asparagus rinds 1, green bean haulms, artichoke by-products (hay and silage) and apple by-products (apple pomace alone or mixed with pear pomace)) were placed to the right along the x-axis. Principal component 2 did not segregate the samples so clearly, although materials with less protein were positioned in the upper part (e.g., citrus and sugar beet pulps), and those with a higher protein content towards the bottom (e.g., lettuce, green beans).

## 4. Discussion

A large variation among the by-products used in this study was observed in chemical composition, in vitro digestibility and rumen fermentation kinetics (rate and extent of degradation). As expected, the wide range detected in chemical composition resulted in large variability in in vitro digestibility and fermentation kinetics. The range of variation in chemical composition among by-products was in line with previous studies [5,14,29,30]. Our results also showed variation in chemical composition for different accessions of the same by-product obtained from different geographical origins (samples of orange pulp or asparagus rinds) or with different conservation techniques (artichoke by-products hay or silage). Aspects such as parent material, origin or conservation methods are important factors that affect the nutritive value of agro-industrial by-products [30].

Multivariate exploratory techniques are valuable methods for data mining used extensively for classification and discrimination purposes. As explained above, by-products were classified by multivariate cluster analysis using all the data from chemical composition, in vitro digestibility and fermentation kinetics. The resultant chart showed that samples of by-products formed two clear clusters, which could be classified in four distinct groups.

Group A included olive tree leaves, pepper skins and grape seeds, which were the least degradable by-products according to the results for in vitro digestibility and fermentation kinetics. Both pepper skins and grape seeds showed the greatest NDF, ADF and ADL contents among all by-products used in the current study. Lignin represented 641 and 509 g/g of the NDF for grape seeds and pepper skins respectively. Due to the high lignification of the cell wall, both by-products showed the lowest volumes of gas production (G24 and *A*, an indicator of the extent of fermentation by mixed ruminal microorganisms) and the lowest digestibility of DM and NDF. The high degree of lignification of grape seeds would explain the very low digestibility of its cell wall (0.064). Although olive tree leaves did not show a very high NDF content, the degree of lignification was rather extreme (437 g ADL/g NDF), explaining its low digestibility, extent of degradation in the rumen (*E*) and volumes of gas produced from in vitro fermentation. Chemical composition of both grape seeds and olive tree leaves is in accordance with previous literature [14,31]. In agreement with the cluster analysis, samples of olive tree leaves, pepper skins and grape seeds were positioned nearby each other in the PCA plot, placed to the right of the *x*-axis (component 1). Considering the weight of each variable on this component, this position along the *x*-axis would correspond to high-fibre low-digestibility and degradability feedstuffs. With these nutritional characteristics, the by-products of this group can be considered of limited nutritive value, and their use in ruminant diets would be restricted to replacing roughage either to increase rumen fill (not a usual situation in modern ruminant farming systems) or to provide insoluble fibre to starchy diets to decrease the risk of ruminal acidosis.

Cluster D included clementine, orange and lemon pulps, as well as dehydrated and ensiled sugar beet pulp. These by-products were the most degradable according to the results for in vitro digestibility and fermentation kinetics. Citrus and sugar beet pulps showed the highest volumes of gas production (G24 and *A* values), and fastest rates of fermentation (*c* and AR). These results indicate that citrus and sugar beet pulps were fermented more extensively and at a faster rate than most of the other by-products used in the study. Moreover, these pulps were the most digestible by-products. Citrus pulps contained less NDF and ADF and thus were more degradable and digestible than sugar beet pulps. Sugar beet pulp is a by-product widely used in ruminant rations. Values of NDF and ADF for sugar beet pulp were in agreement with the literature [32], confirming this is a feedstuff rich in soluble fibre. On the other hand, citrus pulps are known for their high soluble carbohydrates and rapidly digested NDF contents [33]. Chemical composition of citrus pulps was similar to those reported by Bampidis and Robinson [33], showing low CP and NDF contents and high NSC. In the current study, all citrus by-products showed similar in vitro digestibility and fermentation kinetics values regardless of the parent fruit, the results being in agreement with previous studies [33,34]. However, both orange pulps showed dissimilar chemical composition (more NDF and ADF and less NSC in CPO2 than in CPO1) and in vitro fermentability and digestibility, since CPO1 was more fermentable (more gas production), was fermented at a faster rate (higher *c* and shorter *t*_1/2_) and was more digestible (IVDMD and IVNDFD) than CPO2. These differences can be attributed to their different geographical origin (parent material) or to the post-harvest industrial processing. All the citrus pulps (clementine, lemon and orange 1 pulps) from the same place showed similar chemical composition, in vitro digestibility and fermentation kinetics, whereas orange pulp 2 differed. These results confirm that variability within the same by-product may occur, which could be attributed to different factors such as origin or processing. According to the cluster analysis, samples of citrus sugar beet pulps were positioned close to each other in the PCA plot. As all the samples present in group D showed high values of in vitro digestibility and GP and low fibre contents, they were positioned on the left side of the *x*-axis (principal component 1). Based on their chemical composition (low fibre, low protein, high NSC), high digestibility and high rumen degradability, citrus and beet pulps included in this group can be considered as by-products with potentially high-energy value, which can be incorporated in ruminant diets as replacements for the feedstuffs that constitute the main source of energy, such as cereal grains.

Groups B and C could be considered closer to group D (most digestible) than to group A, with a level of inter-group similarity for groups B, C and D of about 55%. By-products classified in group C contained less fibre (NDF and ADF) and were more fermentable and digestible (greater G24, *A*, IVDMD, IVNDFD, D144 and *E* and faster fermentation rate) than those in group B. This variation in chemical composition, in vitro digestibility and fermentation kinetics may explain the discrimination between both groups in the dendrogram derived from the multivariate cluster analysis, and the position of the by-products of each group in the PCA chart. It is noteworthy that accessions of some by-products from the same plant species were classified in divergent groups. For instance, asparagus rinds 1 was ascribed to group B, whereas the sample 2 was assigned to group C, due to the different fibre contents (sample 1 was more fibrous than sample 2) and digestibility (asparagus 2 was more digestible than asparagus 1, with values approximating those of group D). For that reason, one accession of asparagus rinds (ASP2) was in group C and the other (ASP1) was in group B. Both were in the lower part of the PCA plot, most likely due to their high protein content (20%–21%). Similarly, green bean haulms alone or mixed with sugar beet pulp were classified into separate groups. The mixture of green bean haulms and sugar beet pulp was less fibrous and more digestible than green bean haulms alone. Therefore, green bean haulms were in group B and the mixture in group C, approaching group D where sugar beet pulp was classified. The by-products with more CP were in groups B and C, as those included in groups A and D were low in protein (except for olive leaves). The by-products with greatest CP contents were asparagus rinds, pea haulms, pepper cores and dried tomato pulp, although many others showed CP contents above 16%. Predictably, pea haulms have a great CP content since pea plants are leguminous [35,36]. According to Aberoumand [37], asparagus presented high values of CP, due to the high protein content of the whole vegetable (327 vs. 226 for whole asparagus vs. asparagus rinds 2). Regarding dried tomato pulp and pepper cores both had seeds, which normally are rich in protein [38,39]. Chemical composition of dried tomato pulp in the current experiment was in agreement with previous studies [30,40,41]. Samples with higher CP content were positioned at the bottom of the PCA plot, and samples with lower CP values were positioned at the top. Furthermore, 8 out of 10 samples of by-products with high CP content were located together, below the axis of principal component 2. Therefore, most of the by-products of groups B and C could be catalogued as of acceptable digestibility (and thus energy value) and in many cases rich in protein. Their inclusion as feedstuffs in ruminant diets would be determined by their cost and availability and by the composition of the diet into which they would be incorporated.

## 5. Conclusions

The by-products used in the current study showed noticeable variation in chemical composition, in vitro digestibility and fermentation kinetics. Multivariate analysis clustered the by-products into four distinctive groups, according to chemical composition, digestibility and fermentation characteristics. Most by-products used in this study can be considered as potential ingredients in ruminant rations. The more degradable by-products showed higher values of in vitro digestibility and an extensive ruminal fermentability, whereas others were less digestible. Due to the discrimination among by-products, they might represent alternatives sources of different feed components. Citrus pulps could be a source of energy, while asparagus rinds or pea haulms could provide protein to the ration. In addition, sugar beet pulp might be a suitable soluble fibre source, whereas olive tree leaves or pepper skins contain less digestible lignified fibre. In conclusion, some by-products could replace, at least partially, the cereals in ruminant diets, while others might potentially replace the protein or the high-fibre roughage.

## Figures and Tables

**Table 1:** By-products and their geographical origin.

By-Product	ID—Abbreviation	Location *
Sugar beet pulp (dehydrated)	SBPd	León
Ensiled sugar beet pulp	SBPs	León
Sugar beet tops and leaves	SBT	León
Sugar beet rootlets, hairs, root tips and beet tails	SBR	León
Grape seeds	GPS	La Rioja
Olive tree leaves	OLV	Murcia
Almond hulls	ALH	Murcia
Broccoli stalk hay	BRH	Murcia
Lettuce leaves	LTL	Murcia
Asparagus rinds_1_	ASP_1_	Navarra
Asparagus rinds_2_	ASP_2_	La Rioja
Green bean haulms	GBH	La Rioja
Mixture green bean haulms + sugar beet pulp	GBB	La Rioja
Artichoke by-products (ensiled)	ARTs	Murcia
Artichoke by-products (hay)	ARTd	Murcia
Pea haulms	PHL	La Rioja
Broad bean haulms	BBH	La Rioja
Dried tomato pulp	TMP	Aragón
Pepper by-products (cores)	PPC	Aragón
Pepper by-products (skins)	PPS	Aragón
Apple pomace	APL	Aragón
Apple and pear pomace	APP	Aragón
Citrus pulp (clementine)	CPC	Murcia
Citrus pulp (lemon)	CPL	Murcia
Citrus pulp (orange)_1_	CPO_1_	Murcia
Citrus pulp (orange)_2_	CPO_2_	Aragón

* Geographic coordinates (latitude and longitude) for each location: León 42° 36′ N 5° 34′ W; La Rioja 42° 28′ N 2° 26′ W; Navarra 42° 36′ N 1° 50′ W; Aragón 41° 23′ N 0° 46′ W; Murcia 37° 59′ N 1° 8′ W.

**Table 2:** Chemical composition (g/kg dry matter) and metabolizable energy concentration (MJ/kg dry matter) of by-products.

By-Product	OM	CP	EE	NDF	ADF	ADL	NSC	ME
Sugar beet pulp (dehydrated)	869	116	3	460	237	40	291	10.1
Ensiled sugar beet pulp	937	110	3	430	246	25	394	10.4
Sugar beet tops and leaves	874	157	15	290	113	27	412	9.7
Sugar beet rootlets, hairs, root tips and beet tails	879	103	4	435	215	87	337	8.0
Grape seeds	960	116	52	682	584	437	110	4.7
Olive tree leaves	842	98	47	529	345	231	168	6.3
Almond hulls	914	53	18	322	224	110	520	8.0
Broccoli stalk hay	899	155	66	556	349	54	122	8.4
Lettuce leaves	823	184	23	224	158	34	392	8.6
Asparagus rinds_1_	912	206	16	455	293	67	235	8.1
Asparagus rinds_2_	941	226	20	305	169	24	390	10.1
Green bean haulms	769	169	34	415	323	136	152	8.9
Mixture green bean haulms + sugar beet pulp	879	152	17	361	231	44	350	9.6
Artichoke by-products (ensiled)	921	126	19	591	404	110	185	7.4
Artichoke by-products (hay)	948	177	41	678	467	76	51	8.2
Pea haulms	902	199	32	282	146	14	389	10.2
Broad bean haulms	902	173	21	335	185	18	373	10.0
Dried tomato pulp	965	190	51	557	427	260	167	7.8
Pepper by-products (cores)	893	192	67	311	222	55	323	8.9
Pepper by-products (skins)	949	99	33	753	641	383	64	4.4
Apple pomace	984	51	60	672	460	150	201	7.4
Apple and pear pomace	987	48	27	683	460	129	229	6.9
Citrus pulp (clementine)	972	73	20	139	96	2	740	11.3
Citrus pulp (lemon)	957	76	77	247	171	3	558	12.6
Citrus pulp (orange)_1_	969	80	26	222	126	2	640	11.7
Citrus pulp (orange)_2_	940	110	25	308	233	14	496	9.1

OM = organic matter; CP = crude protein; EE = ether extract; NDF = neutral detergent fibre; ADF = acid detergent fibre; ADL = acid detergent lignin; NSC = non-structural carbohydrates; ME = metabolizable energy (calculated following the equation of Menke and Steingass [25]).

**Table 3:** Rumen fermentation kinetics estimated from in vitro gas production profiles of the by-products.

By-Product	*A*	*c*	Lag	G24	AR	*t*_1/2_	PF
Sugar beet pulp (dehydrated)	340	0.081	3.77	273	13.8	12.4	2.56
Ensiled sugar beet pulp	356	0.074	2.62	283	14.9	12.0	2.56
Sugar beet tops and leaves	297	0.075	1.72	241	13.5	11.0	3.06
Sugar beet rootlets, hairs, root tips and beet tails	300	0.057	3.30	208	9.7	15.4	2.85
Grape seeds	78	0.065	0.00	62	3.7	10.7	2.89
Olive tree leaves	152	0.042	0.93	94	4.4	17.4	3.11
Almond hulls	263	0.063	1.12	201	10.9	12.1	2.96
Broccoli stalk hay	202	0.058	0.27	151	8.3	12.2	3.39
Lettuce leaves	231	0.074	2.46	186	10.4	11.8	4.06
Asparagus rinds_1_	216	0.060	0.83	162	8.7	12.4	3.33
Asparagus rinds_2_	300	0.068	1.22	236	13.1	11.4	3.13
Green bean haulms	252	0.067	2.20	193	10.0	12.6	3.22
Mixture green bean haulms + sugar beet pulp	294	0.075	1.90	238	13.2	11.1	3.05
Artichoke by-products (ensiled)	226	0.058	1.30	156	7.9	13.3	3.49
Artichoke by-products (hay)	275	0.043	4.60	156	6.6	20.7	2.88
Pea haulms	284	0.080	0.89	240	14.9	9.5	2.78
Broad bean haulms	293	0.080	0.71	247	15.6	9.4	3.01
Dried tomato pulp	200	0.065	1.62	153	8.1	12.3	2.72
Pepper by-products (cores)	219	0.081	0.74	186	11.8	9.3	3.57
Pepper by-products (skins)	129	0.036	2.77	69	2.9	22.0	2.81
Apple pomace	218	0.070	1.80	171	9.3	11.7	2.40
Apple and pear pomace	228	0.061	2.12	168	8.4	13.5	2.39
Citrus pulp (clementine)	368	0.082	1.53	309	18.4	10.0	2.66
Citrus pulp (lemon)	350	0.090	1.31	303	19.2	9.0	2.78
Citrus pulp (orange)_1_	374	0.082	1.70	314	18.5	10.1	2.62
Citrus pulp (orange)_2_	322	0.054	1.88	224	10.9	14.8	2.94

*A* = asymptotic gas production (mL/g DM incubated); *c* = fractional rate of fermentation (h^−1^); Lag = lag time (h); G24 = volume of fermentation gas produced at 24 h incubation (mL/g DM incubated); AR = average gas production rate (mL /g DM per h); *t*_1/2_ = time (h) for a gas production of A/2; PF = partitioning factor (mg DM digested/mL fermentation gas).

**Table 4:** In vitro digestibility and extent of ruminal degradation of the by-products.

By-Product	IVDMD	IVNDFD	D144	*E*
Sugar beet pulp (dehydrated)	0.929	0.845	0.870	0.542
Ensiled sugar beet pulp	0.940	0.860	0.910	0.575
Sugar beet tops and leaves	0.957	0.851	0.904	0.589
Sugar beet rootlets, hairs, root tips and beet tails	0.873	0.707	0.854	0.483
Grape seeds	0.362	0.064	0.227	0.150
Olive tree leaves	0.745	0.518	0.469	0.254
Almond hulls	0.840	0.502	0.778	0.491
Broccoli stalk hay	0.764	0.575	0.686	0.431
Lettuce leaves	0.967	0.851	0.900	0.572
Asparagus rinds_1_	0.836	0.640	0.687	0.428
Asparagus rinds_2_	0.960	0.868	0.919	0.591
Green bean haulms	0.859	0.660	0.809	0.501
Mixture green bean haulms + sugar beet pulp	0.934	0.817	0.894	0.581
Artichoke by-products (ensiled)	0.759	0.592	0.723	0.432
Artichoke by-products (hay)	0.817	0.730	0.791	0.382
Pea haulms	0.893	0.620	0.786	0.539
Broad bean haulms	0.927	0.784	0.877	0.604
Dried tomato pulp	0.683	0.431	0.544	0.340
Pepper by-products (cores)	0.852	0.523	0.779	0.539
Pepper by-products (skins)	0.432	0.245	0.363	0.172
Apple pomace	0.566	0.355	0.523	0.333
Apple and pear pomace	0.599	0.412	0.544	0.328
Citrus pulp (clementine)	0.991	0.938	0.976	0.658
Citrus pulp (lemon)	0.975	0.898	0.956	0.667
Citrus pulp (orange)_1_	0.989	0.949	0.979	0.658
Citrus pulp (orange)_2_	0.966	0.890	0.944	0.546

IVDMD = in vitro DM digestibility (g/g DM incubated); IVNDFD = in vitro NDF digestibility (g/g NDF incubated); DM144 = DM disappearance after 144 h of incubation (g/g DM incubated); *E* = extent of degradation in the rumen for a rate of passage of 0.033 h^−1^ (g DM degraded/g DM ingested).
